# Benefit and Risk of Prolonged Dual Antiplatelet Therapy After Percutaneous Coronary Intervention With Drug-Eluting Stents in Patients With Elevated Lipoprotein(a) Concentrations

**DOI:** 10.3389/fcvm.2021.807925

**Published:** 2021-12-20

**Authors:** Kongyong Cui, Hao-Yu Wang, Dong Yin, Chenggang Zhu, Weihua Song, Hongjian Wang, Lei Jia, Dong Zhang, Chenxi Song, Lei Feng, Kefei Dou

**Affiliations:** ^1^Cardiometabolic Medicine Center, Department of Cardiology, Fuwai Hospital, National Center for Cardiovascular Diseases, Chinese Academy of Medical Sciences and Peking Union Medical College, Beijing, China; ^2^Coronary Heart Disease Center, Department of Cardiology, Fuwai Hospital, National Center for Cardiovascular Diseases, Chinese Academy of Medical Sciences and Peking Union Medical College, Beijing, China; ^3^State Key Laboratory of Cardiovascular Disease, Beijing, China; ^4^National Clinical Research Center for Cardiovascular Diseases, Beijing, China

**Keywords:** lipoprotein(a) [Lp(a)], coronary artery disease, percutaneous coronary intervention (or PCI), drug-eluting stent (DES), DAPT (dual antiplatelet therapy), clinical outcome

## Abstract

**Background:** Lipoprotein(a) is positively related to cardiovascular events in patients with coronary artery disease (CAD). Given that lipoprotein(a) has a prothrombotic effect, prolonged dual antiplatelet therapy (DAPT) might have a beneficial effect on reducing ischemic events in patients with elevated lipoprotein(a) levels after percutaneous coronary intervention (PCI). We performed this study to assess the efficacy and safety of prolonged DAPT (>1 year) in this population.

**Methods:** We evaluated a total of 3,025 CAD patients with elevated lipoprotein(a) levels who were event-free at 1 year after PCI from the prospective Fuwai PCI Registry, of which 913 received DAPT ≤ 1 year and 2,112 received DAPT>1 year. The primary endpoint was major adverse cardiovascular and cerebrovascular event (MACCE), defined as a composite of all-cause death, myocardial infarction or stroke.

**Results:** After a median follow-up of 2.4 years, patients who received DAPT>1 year were associated with lower risks of MACCE compared with DAPT ≤ 1 year (1.6 vs. 3.8%; hazard ratio [HR] 0.383, 95% confidence interval [CI] 0.238–0.616), which was primarily driven by the lower all-cause mortality (0.2 vs. 2.3%; HR 0.078, 95% CI 0.027–0.227). In addition, DAPT>1 year was also associated with lower risks of cardiac death, and definite/probable stent thrombosis than those who received DAPT ≤ 1 year (*P* < 0.05). Conversely, no difference was found between the two groups in terms of clinically relevant bleeding. Similar results were observed in multivariate Cox regression analysis and inverse probability of treatment weighting analysis.

**Conclusions:** In patients with elevated lipoprotein(a) concentrations after PCI, prolonged DAPT (>1 year) reduced ischemic cardiovascular events, including MACCE, all-cause mortality, cardiac mortality, and definite/probable stent thrombosis, without increase in clinically relevant bleeding risk compared with ≤ 1-year DAPT. Lipoprotein(a) levels might be a new important consideration when deciding the duration of DAPT after PCI.

## Introduction

Lipoprotein(a) [Lp(a)] is a low-density lipoprotein (LDL)-like particle in which an apolipoprotein(a) [apo(a)] covalently linked to its apolipoprotein B100 component via a disulfide bridge (1–4). During the past decades, plasma Lp(a) has been recognized as a novel risk factor for the incidence of cardiovascular disease (5–11). Furthermore, an increasing evidence has supported that Lp(a) levels play an important role in predicting subsequent ischemic events in patients with established coronary artery disease (CAD), especially those who underwent percutaneous coronary intervention (PCI) (12–16). In a multicenter and prospective study, Liu et al. demonstrated that high Lp(a) levels were positively associated with adverse cardiovascular events in 4,078 stable CAD patients after PCI at a mean follow-up of 4.9 years (15). Nevertheless, there are still no approved pharmacologic therapies that specifically target high Lp(a) levels. Recently, as a novel therapeutic agent, hepatocyte-directed antisense oligonucleotide APO(a)-LRx has been proven to reduce Lp(a) levels by 80% (17). However, the effect of this Lp(a)-lowering drug on adverse cardiovascular events remains unknown.

Dual antiplatelet therapy (DAPT) consisting of aspirin and a P2Y_12_ receptor inhibitor represents the cornerstone of pharmacological treatment aimed at preventing thrombotic complications after PCI. Considering that Lp(a) has a prothrombotic effect through its inactive, plasminogen-like protease domain on apo(a) (2, 4), we speculate that prolonged DAPT may have a beneficial effect on reducing future ischemic events in patients who had elevated Lp(a) levels after PCI. However, the relative benefit of prolonged DAPT in this high-risk population has never been assessed. We therefore performed this study to compare the outcomes of prolonged DAPT (> 1 year) vs. shortened DAPT (≤ 1 year) in patients with elevated Lp(a) levels who were event-free at 1 year after PCI with drug-eluting stent (DES) in a large and contemporary PCI registry.

## Materials and Methods

### Study Design and Population

This was a retrospective analysis of a single-center, prospective registry and the study design has been previously described (18–20). Patients with CAD and elevated Lp(a) levels who underwent PCI with DES at Fuwai Hospital, National Center for Cardiovascular Diseases between January 2013 through December 2013 were consecutively enrolled. The study was performed according to the ethical guidelines of the 1975 Declaration of Helsinki and the study protocol has been priorly approved by the ethical committee of Fuwai Hospital, National Center for Cardiovascular Diseases. All the patients provided written informed consent before enrollment. Previous meta-analyses and the current guidelines for the management of dyslipidemia from China and Canada suggested that the relation between Lp(a) and cardiovascular risk inflects at a concentration of 30 mg/dl (8, 14, 21, 22). We therefore used a cut-off of >30 mg/dl to assign abnormal Lp(a) levels. For the present analysis, patients who had missing Lp(a) data (*n* = 665) or normal Lp(a) concentrations (*n* = 6,564), had follow-up duration ≤ 1 year (*n* = 35) were not included. We also excluded patients who did not use DES (*n* = 137) or experienced adverse cardiovascular events (death, myocardial infarction [MI], stent thrombosis[ST], stroke, repeat revascularization, or Bleeding Academic Research Consortium [BARC] type 2, 3, or 5 bleeding) within 1-year follow-up (*n* = 298). Finally, 3,025 patients who met the selection criteria were divided into 2 groups according to the DAPT (aspirin plus clopidogrel) duration, i.e., DAPT >1-year group and DAPT ≤ 1-year group. Landmark analysis was conducted to classify patients into treatment groups based on 1-year antiplatelet treatment after PCI and to evaluate prognosis from the landmark time point.

### Study Procedures and Biochemical Analysis

All the procedures and medical therapies were performed in compliance with guidelines' recommendation and operators' discretion. Detailed information on procedures has been previously described (19, 20). After an overnight fasting, venous blood samples for measurement of Lp(a) and other biomarkers were obtained from all patients, and the test was conducted through clinical chemistry department in central lab of our hospital. Lp(a) was measured by immunoturbidimetry method [LASAY Lp(a) auto; SHIMA Laboratories Co., Ltd, Tokyo, Japan] with a normal value of <30 mg/dl. Levels of low-density lipoprotein cholesterol (LDL-C), high-density lipoprotein cholesterol, and total cholesterol were analyzed using the automated biochemical analyzer (Hitachi 7150, Tokyo, Japan), and glycosylated hemoglobin was tested with the Tosoh Automated Glycohemoglobin Analyser (HLC-723G8; Tosoh Corporation, Tokyo, Japan).

### Follow-Up and Endpoints

Demographics, cardiovascular risk factors, clinical parameters, laboratory data, angiographic, and procedural details were prospectively collected in our dedicated PCI registry by independent research personnel. After index PCI, patients were followed up at 1, 6, and 12 months and annually thereafter. Data for endpoints were collected from medical records, clinical visit, and telephone interviews by trained investigators who were blind to the clinical data. To record ≥ 2-year follow-up information, the follow-up period was extended to January 31, 2016 for the present study. Adherence to antiplatelet medication was routinely assessed at each time of follow-up, and the status of antiplatelet therapy was collected by dedicated questionnaires and the electronic prescribing system at our center.

The primary endpoint was major adverse cardiovascular and cerebrovascular event (MACCE), defined as a composite of all-cause death, non-fatal MI, or stroke. Secondary endpoints included the individual components of the primary endpoint, cardiac death, definite or probable ST, and BARC type 2, 3, or 5 bleeding. Deaths were classified as either cardiac or non-cardiac. All deaths were considered to be cardiac-related unless a non-cardiac origin was documented. MI was defined according to the third universal definition of MI (23). Stoke was defined as new focal neurological deficit lasting > 24 h, which confirmed by a neurologist based on imaging evidence. Definite or probable ST was adjudicated on the basis of the Academic Research Consortium criteria (24). Bleeding events were categorized on the basis of the BARC classifications (25). Moreover, all the events were carefully verified and adjudicated by independent clinicians.

### Statistical Analysis

Continuous variables were expressed as mean ± standard deviation and categorical variables were expressed as frequencies (percentages). Differences in various characteristics were compared using Student's t test, Wilcoxon's rank sum test, Pearson's chi-square test, and Fisher's exact test, when appropriate. Cumulative incidence of clinical events was estimated using Kaplan-Meier curves, and differences were assessed with log-rank test. Univariate and multivariate Cox regression analyses were performed to calculate hazard ratios (HRs) and 95% confidence intervals (CIs). Variables in Table 1 with *P* < 0.05 in univariate analysis or those that were clinically relevant were entered into the multivariable model, including age, sex, body mass index, current smoker, diabetes, hypertension, dyslipidemia, previous MI, previous PCI, previous coronary artery bypass graft surgery, previous stroke, peripheral vascular disease, acute coronary syndrome (ACS), total cholesterol, LDL-C, baseline SYNTAX score, multivessel disease, total lesion length, bifurcation lesion, in-stent restenosis, minimum stent diameter, total stent length, and use of statin at discharge.

**Table 1:** Baseline patient, angiographic, and procedural characteristics according to DAPT time.

**Variable**	**DAPT ≤ 1 year (*n =* 913)**	**DAPT > 1 year (*n =* 2,112)**	***P*-value**
Age, years	58.6 ± 10.1	58.8 ± 10.0	0.850
Male, *n* (%)	689 (75.5)	1,542 (73.0)	0.159
Body mass index, kg/m^2^	25.6 ± 3.1	25.8 ± 3.2	0.409
Current smoker, *n* (%)	515 (56.4)	1,120 (53.0)	0.087
Diabetes mellitus, *n* (%)	266 (29.1)	603 (28.6)	0.745
Hypertension, *n* (%)	579 (63.4)	1,384 (65.5)	0.264
Dyslipidemia, *n* (%)	584 (64.0)	1,450 (68.7)	0.012
Previous myocardial infarction, *n* (%)	161 (17.6)	419 (19.8)	0.157
Previous PCI, *n* (%)	214 (23.4)	495 (23.4)	0.999
Previous CABG, *n* (%)	39 (4.3)	103 (4.9)	0.470
Previous stroke, *n* (%)	102 (11.2)	231 (10.9)	0.850
Peripheral vascular disease, *n* (%)	19 (2.1)	59 (2.8)	0.256
Chronic kidney disease, *n* (%)	86 (9.4)	233(11.0)	0.185
COPD, *n* (%)	30 (3.3)	55 (2.6)	0.298
LVEF, %	63.0 ± 7.5	62.9 ± 7.3	0.691
LVEF <50%, *n* (%)	45 (5.1)	107 (5.2)	0.869
Acute coronary syndrome, *n* (%)	568 (62.2)	1,205 (57.1)	0.008
Systolic blood pressure, mmHg	127.1 ± 17.8	126.5 ± 17.1	0.379
Laboratory data
WBC, 10^3^/uL	6.76 ± 1.66	6.71 ± 1.64	0.630
Hemoglobin, g/L	142.5 ± 15.3	142.3 ± 15.7	0.733
Total cholesterol, mmol/L	4.33 ± 1.04	4.36 ± 1.09	0.634
LDL-C, mmol/L	2.65 ± 0.90	2.66 ± 0.93	0.882
HDL-C, mmol/L	1.06 ± 0.28	1.06 ± 0.28	0.798
HbA1c, %	6.58 ± 1.28	6.62 ± 1.22	0.123
Lp(a), mg/dL	60.9 ± 24.9	60.9 ± 24.5	0.873
Radial artery access, *n* (%)	775 (92.4)	1,758 (90.4)	0.093
Multivessel disease, *n* (%)	682 (74.7)	1,629 (77.1)	0.148
SYNTAX score	12.5 ± 7.9	12.3 ± 7.8	0.593
SYNTAX score >22, *n* (%)	104 (11.8)	249 (12.3)	0.720
Total lesion length, mm	38.9 ± 25.1	39.9 ± 26.7	0.534
Target lesion morphology
Bifurcation lesion, *n* (%)	192 (21.0)	429 (20.3)	0.654
2-stent technique, *n* (%)	37 (4.1)	98 (4.6)	0.472
Chronic total occlusion, *n* (%)	167 (18.3)	421 (19.9)	0.295
In-stent restenosis, *n* (%)	39 (4.3)	96 (4.5)	0.738
Severe calcification, *n* (%)	27 (3.0)	67 (3.2)	0.754
Angulation > 45 degrees, *n* (%)	99 (10.8)	235 (11.1)	0.819
Type B2 or C lesion, *n* (%)	708 (77.5)	1,655 (78.4)	0.619
No. vessels treated	1.30 ± 0.51	1.28 ± 0.50	0.321
No. lesions treated	1.45 ± 0.67	1.44 ± 0.68	0.485
No. lesions treated ≥3, *n* (%)	69 (7.6)	146 (6.9)	0.526
Drug-eluting stent number	1.91 ± 1.02	1.96 ± 1.06	0.344
Drug-eluting stent number ≥ 3, *n* (%)	220 (24.1)	502 (23.8)	0.846
Type of drug-eluting stent			0.531
PES/SES, *n* (%)	412 (45.1)	927 (43.9)	
EES/ZES, *n* (%)	501 (54.9)	1,185 (56.1)	
Minimum stent diameter, mm	2.91 ± 0.49	2.90 ± 0.49	0.524
Total stent length, mm	42.9 ± 25.9	43.7 ± 27.2	0.534
DAPT score	1.61 ± 1.25	1.56 ± 1.23	0.300
DAPT score≥2, *n* (%)	498 (54.5)	1,128 (53.4)	0.565
Medications at discharge
Aspirin, *n* (%)	900 (98.6)	2,088 (98.9)	0.509
Clopidogrel, *n* (%)	901 (98.7)	2,074 (98.2)	0.337
β-blockers, *n* (%)	828 (90.7)	1,931 (91.4)	0.509
Statins, *n* (%)	879 (96.3)	,2031 (96.2)	0.883
Calcium channel blockers, *n* (%)	442 (48.4)	1,041 (49.3)	0.657
DAPT time, days	350 ± 56	666 ± 166	<0.001

*CABG, coronary artery bypass grafting; COPD, chronic obstructive pulmonary disease; DAPT, dual antiplatelet therapy; EES, everolimus-eluting stent; HDL-C, high-density lipoprotein cholesterol; LDL-C, low-density lipoprotein cholesterol; LVEF, left ventricular ejection fraction; PES, paclitaxel-eluting stent; PCI, percutaneous coronary intervention; SES, sirolimus-eluting stent; WBC, white blood cell; ZES, zotarolimus-eluting stent*.

Notably, inverse probability of treatment weighting (IPTW) analysis was also conducted to adjust for differences in baseline characteristics to evaluate the relative efficacy and safety of DAPT >1 year vs. DAPT ≤ 1 year in patients with elevated Lp(a) levels. The propensity score was calculated using a non-parsimonious multivariable logistic regression model and considering DAPT time (>1 year vs. ≤ 1 year) as dependent variable. Covariates used for the propensity score model included age, sex, body mass index, current smoker, diabetes, hypertension, dyslipidemia, previous MI, previous PCI, previous stroke, peripheral vascular disease, chronic obstructive pulmonary disease, total cholesterol, LDL-C, total lesion length, type B2 or C lesion, chronic total occlusion (CTO), bifurcation lesion, number of lesions treated, stent number, use of everolimus- or zotarolimus-eluting stent, and use of aspirin, P2Y_12_ inhibitor, β-blocker, and statin at discharge. The detailed methods of IPTW analysis were previously described (26).

Furthermore, subgroup analysis of MACCE was performed based on the following factors, including age (≤ 65 and > 65 years), sex, current smoking, diabetes, previous MI, chronic kidney disease (CKD), clinical presentation (stable angina vs. ACS), diseased vessels, type of DES, and DAPT score. All statistical analyses were conducted with SPSS version 23.0 (SPSS Inc., Chicago, IL, USA) and R version 3.6.0 (R Foundation for Statistical Computing, Vienna, Austria). A two-sided *p* < 0.05 was considered statistically significant.

## Results

### Baseline Characteristics

**Figure 1:**
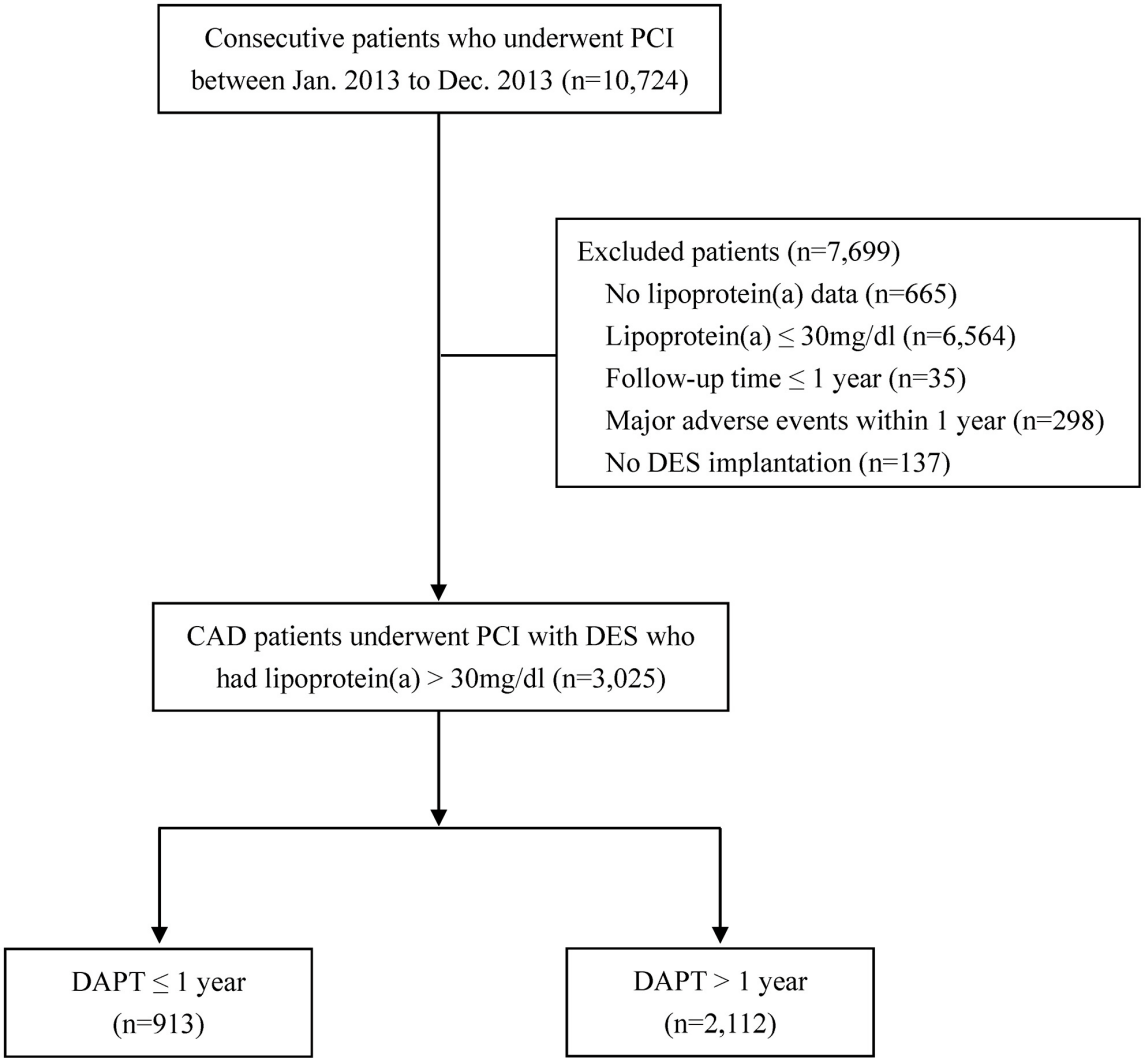
Flow chart of the study. CAD, coronary artery disease; DAPT, dual antiplatelet therapy; DES, drug-eluting stent; PCI, percutaneous coronary intervention.

Among the 3,025 patients with elevated Lp(a) concentrations (>30 mg/dl) who were event-free at 1 year after the index PCI, 913 received DAPT ≤ 1 year and 2,112 received DAPT >1 year (Figure 1). The study participants had an average Lp(a) concentration of 60.9 mg/dl, an average age of 58.7 years, and 73.8% of which were male. In addition, a large number of participants were considered to have traditional cardiovascular risk factors including hypertension (64.9%), dyslipidemia (67.2%), diabetes (28.7%), and current smoking (54.0%). Baseline patient, angiographic and procedural characteristics were similar between the two groups, except for the history of dyslipidemia and clinical presentation of acute coronary syndrome (Table 1). The median follow-up period was 2.4 (2.2–2.6) years.

### Unadjusted Outcomes

**Figure 2:**
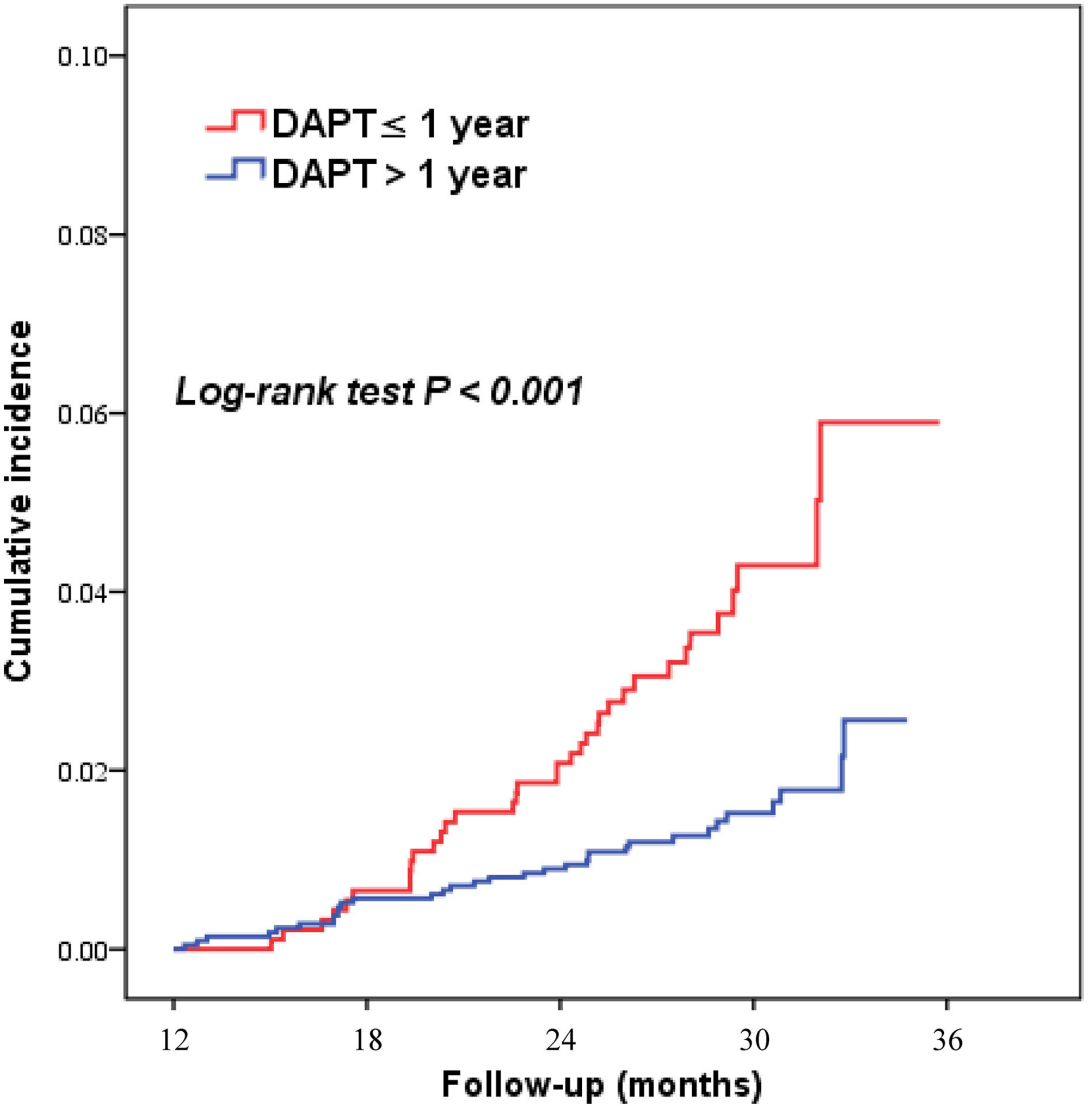
Kaplan–Meier curves for major adverse cardiovascular and cerebrovascular events according to DAPT duration (>1 year vs. ≤ 1 year) in patients with elevated Lp(a) levels. DAPT, dual antiplatelet therapy.

**Figure 3:**
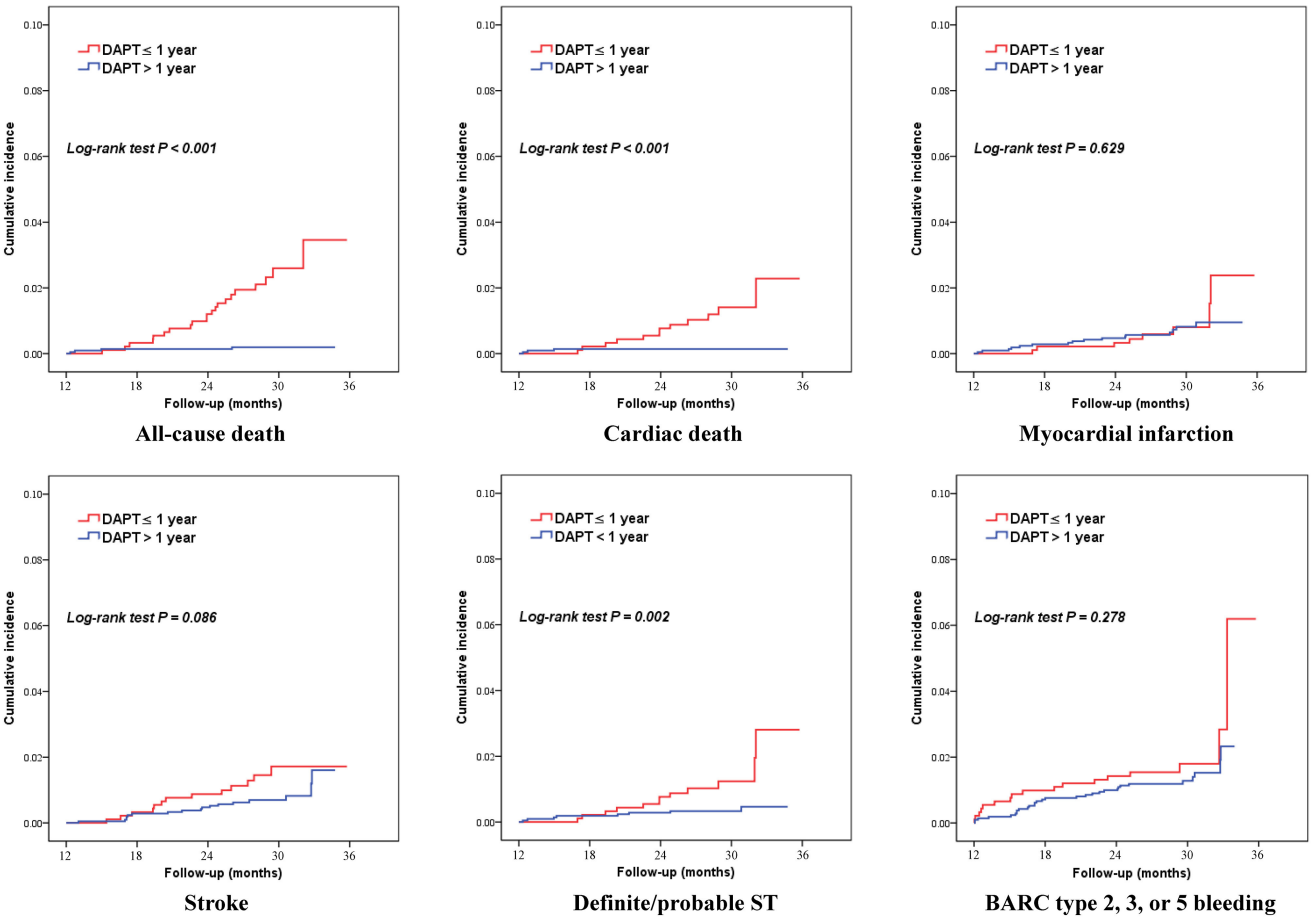
Kaplan–Meier curves for secondary outcomes according to DAPT duration (>1 year vs. ≤ 1 year) in patients with elevated Lp(a) levels. BARC, Bleeding Academic Research Consortium; DAPT, dual antiplatelet therapy; ST, stent thrombosis.

As shown in Table 2, Figures 2, 3, patients who received DAPT >1 year had lower risks of MACCE (1.6 vs. 3.8%; HR 0.383, 95% CI 0.238–0.616), which was primarily caused by the lower all-cause mortality (0.2 vs. 2.3%; HR 0.078, 95% CI 0.027–0.227). In addition, DAPT >1 year was also associated with lower risks of cardiac death (0.1 vs. 1.3%; HR 0.103, 95%CI 0.029–0.366), and definite/probable ST (0.4 vs. 1.3%; HR 0.270, 95%CI 0.110–0.662) than those who received DAPT ≤ 1 year. Interestingly, no difference was found between the two groups in terms of BARC type 2, 3, or 5 bleeding (1.4 vs. 1.9%; HR 0.720, 95%CI 0.397–1.307) at 2.4 years.

**Table 2:** Clinical outcomes at 2.4 years according to DAPT time.

**Clinical endpoint**	**No. patients with event**, ***n*** **(%)**		**Crude HR (95% CI)**	**Multivariate adjusted HR (95% CI)**	**IPTW adjusted HR (95% CI)**
	**DAPT ≤ 1 year**	**DAPT > 1 year**			
All-cause death/MI/stroke	35 (3.8)	33 (1.6)	0.383 (0.238–0.616)	0.335 (0.202–0.555)	0.373 (0.231–0.601)
All–cause death	21 (2.3)	4 (0.2)	0.078 (0.027–0.227)	0.056 (0.016–0.191)	0.076 (0.026–0.223)
Cardiac death	12 (1.3)	3 (0.1)	0.103 (0.029–0.366)	0.056 (0.012–0.263)	0.098 (0.027–0.352)
Non–fatal MI	8 (0.9)	16 (0.8)	0.811 (0.347–1.898)	0.590 (0.241–1.445)	0.770 (0.323–1.836)
Stroke	13 (1.4)	17 (0.8)	0.536 (0.260–1.105)	0.510 (0.239–1.089)	0.514 (0.249–1.059)
Definite/probable ST	12 (1.3)	8 (0.4)	0.270 (0.110–0.662)	0.173 (0.063–0.474)	0.253 (0.102–0.625)
BARC type 2, 3, or 5 bleeding	17 (1.9)	30 (1.4)	0.720 (0.397–1.307)	0.663 (0.359–1.225)	0.754 (0.415–1.372)

*BARC, Bleeding Academic Research Consortium; CI, confidence interval; DAPT, dual antiplatelet therapy; HR, hazard ratio; IPTW, inverse probability of treatment weighting; MI, myocardial infarction; ST, stent thrombosis*.

### Cox Proportional Hazards Regression Analysis

After the potential confounders were adjusted, prolonged DAPT (> 1 year) remained associated with a reduced risk of MACCE (HR _adjusted_ 0.335, 95% CI 0.202–0.555) at 2.4 years. Furthermore, prolonged DAPT was also a significant predictor of lower all-cause mortality (HR _adjusted_ 0.056, 95% CI 0.016–0.191), cardiac mortality (HR _adjusted_ 0.056, 95% CI 0.012–0.263), and definite/probable ST (HR _adjusted_ 0.173, 95% CI 0.063–0.474) (Table 2).

### IPTW Analysis

**Figure 4:**
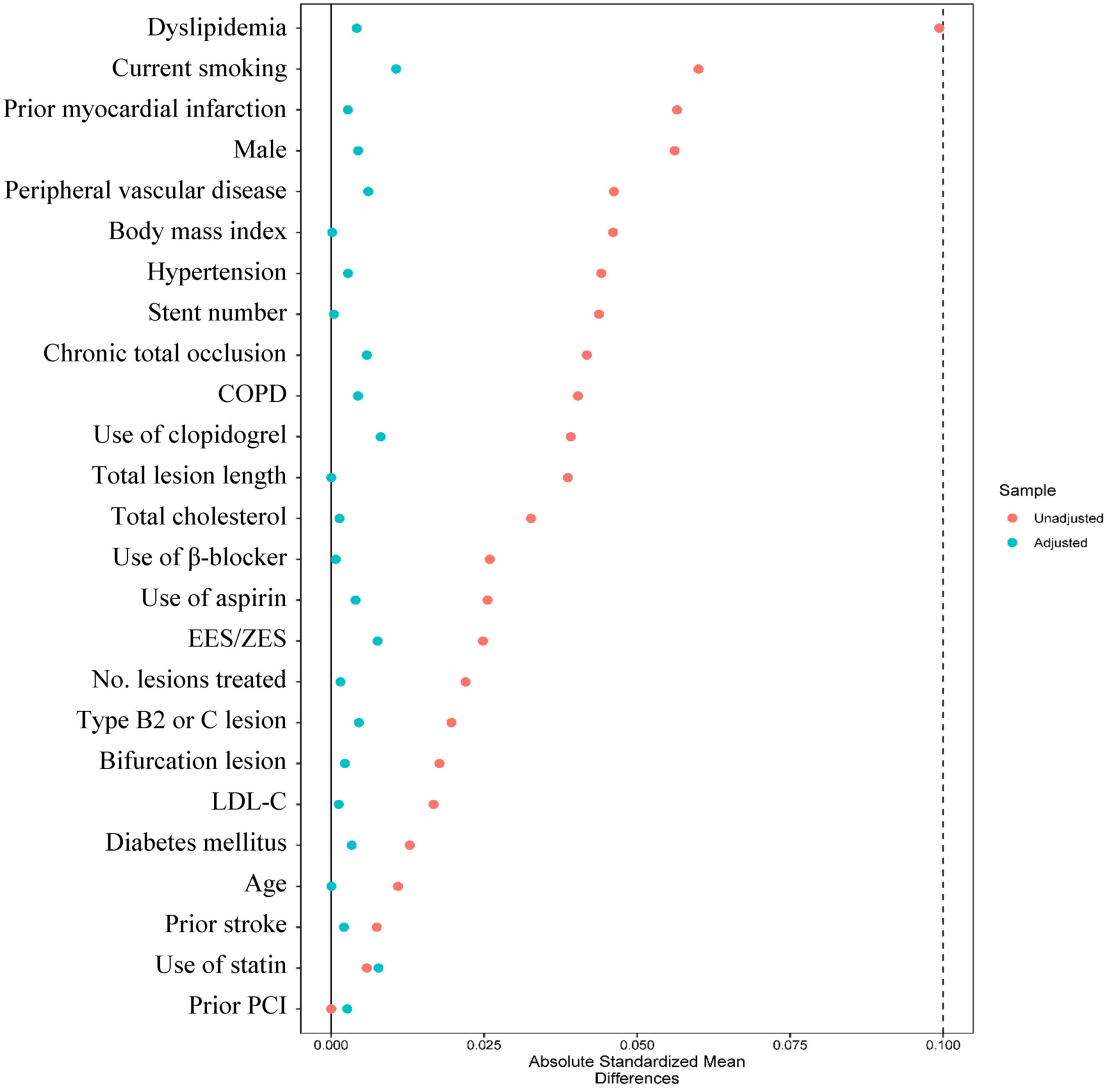
Absolute standard difference before and after inverse probability of treatment weighting analysis between DAPT >1-year and DAPT ≤ 1-year groups. COPD, chronic obstructive pulmonary disease; EES, everolimus-eluting stent; LDL-C, low-density lipoprotein cholesterol; PCI, percutaneous coronary intervention; ZES, zotarolimus-eluting stent.

After IPTW adjustment, all the candidate variables were well-balanced between DAPT > 1-year and DAPT ≤ 1-year groups, with absolute standardized differences <10% (Figure 4). The IPTW analysis obtained consistent results that the risks of MACCE (HR _IPTW_ 0.373, 95% CI 0.231–0.601), all-cause death (HR _IPTW_ 0.076, 95%CI 0.026–0.223), cardiac death (HR _IPTW_ 0.098, 95% CI 0.027 to 0.352), and definite/probable ST (HR_IPTW_ 0.253, 95% CI 0.102–0.625) were significantly decreased in the prolonged DAPT group, and the risk of clinically relevant bleeding was not significantly different between the two antiplatelet therapies (HR _IPTW_ 0.754, 95% CI 0.415–1.372) (Table 2).

### Subgroup Analysis

**Figure 5:**
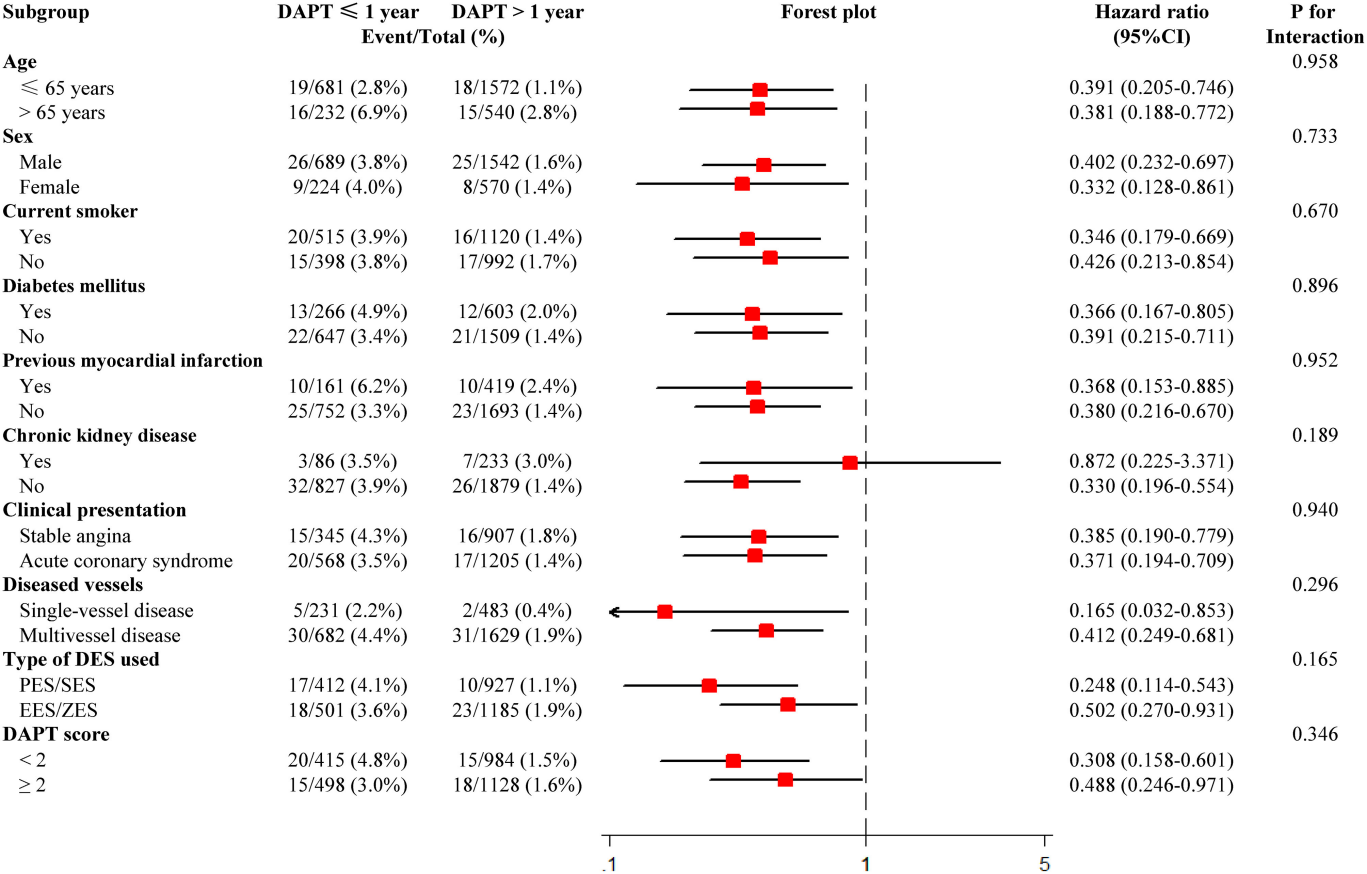
Subgroup analysis for major adverse cardiovascular and cerebrovascular events. CI, confidence interval; DAPT, dual antiplatelet therapy; DES, drug-eluting stent; EES, everolimus-eluting stent; PES, paclitaxel-eluting stent; SES, sirolimus-eluting stent; ZES, zotarolimus-eluting stent.

Subgroup analyses were performed based on important baseline information, and formal testing for interactions indicated that results of the comparison of MACCE between the two groups were consistent across all the subgroups. The lower risk of MACCE in DAPT > 1-year group than DAPT ≤ 1-year group was consistently observed, regardless of age, gender, smoking status, diabetes, history of MI, CKD, clinical presentation, number of diseased vessels, type of DES, and DAPT score (Figure 5).

## Discussion

To the best our knowledge, this is the first study to evaluate the efficacy and safety of prolonged DAPT for CAD patients who had elevated Lp(a) concentrations after PCI with DES. We found that prolonged DAPT (>1 year) was associated with lower risks of MACCE, all-cause mortality, cardiac mortality, and definite/probable ST, without increasing the risk of clinically relevant bleeding at 2.4 years. Furthermore, these favorable prognostic findings for DAPT >1-year against DAPT ≤ 1-year were consistent across all the important clinical and procedural subgroups.

Plasma Lp(a) was initially described in 1963 by Berg K, and it has been recognized as a novel risk factor for cardiovascular disease in recent years. In 2008, Kamstrup et al. reported a stepwise increase in MI with increasing Lp(a) levels in 9,330 general participants from the Copenhagen City Heart Study (6). Henceforth, mounting evidence from meta-analyses, Mendelian randomization studies and genome-wide association studies indicated that Lp(a) was an independent, genetic, and causal risk factor for CAD (5, 7–11). Recently, some studies have indicated that Lp(a) levels was significantly associated with long-term adverse cardiovascular events in patients after PCI (13, 15). A study with 1,768 patients who received statin therapy after PCI showed that elevated Lp(a) levels were associated with increased cardiac death or ACS during a median follow-up of 4.4 years (HR _adjusted_ 1.28, 95%CI 1.04–1.58) (13). In addition, Liu et al. reported that high Lp(a) levels was associated with higher incidence of a composite of cardiac death, MI or stroke in stable CAD patients treated with statins after PCI at 4.9-year follow-up (15).

Nevertheless, there are still no approved pharmacologic therapies that specifically aimed to reduce Lp(a) levels. Evidence showed that the widely used statins have no Lp(a) lowering effect, and has even indicated a slight Lp(a) increasing effect. Though having a 20~30% Lp(a)-lowering effects, both niacin and mipomersen are associated with side effects, and mipomersen is only approved in homozygous familial hypercholesterolemia due to hepatotoxicity (2). PCSK9 inhibitor has been proved to reduce cardiovascular events independent of LDL-C in ACS patients by lowering Lp(a) levels, yet it could only predict a weak reduction in ischemic events (27). Actually, several Mendelian randomization analyses speculated that large absolute reduction in Lp(a) of ~66–100 mg/dl may be required to achieve equivalent protective effects yielded from a 39-mg/dL (or 1-mmol/L) reduction of LDL-C (28, 29). Tsimikas et al. found that a novel therapeutic agent, i.e., APO(a)-L_Rx_, provides potent reductions in levels of Lp(a) in patients with cardiovascular disease by reducing the production of apo(a) which offers greater specificity compared with PCSK9 inhibitor (17). Nonetheless, further trials are in demand to assess the impact of Lp(a) lowering with APO(a)-L_Rx_ on major cardiac events in patients with established CAD.

Lp(a) consists of a LDL-like particle and an apo(a), which bounds to apolipoprotein B100 via a disulfide bond. It is generally believed that Lp(a) may contribute to cardiovascular disease by proatherogenic effects of the LDL-like component, proinflammatory effects of the oxidized phospholipid, and prothrombotic effects of the plasminogen-like apo(a) (1–4). For unknown etiological and physiological reasons, apo(a) has evolved from the plasminogen gene through duplication and remodeling. Due to the similarity between the apo(a) component of Lp(a) and plasminogen, Lp(a) promotes thrombotic and fibrinolytic events through several mechanisms, including inflammation through its content of oxidized phospholipids, the presence of lysine binding sites that allow accumulation in the arterial wall, and potential antifibrinolytic roles by inhibiting plasminogen activation (2, 4). Therefore, intensified antithrombotic therapy may have positive effect on patients with elevated Lp(a) levels after PCI. In this setting, it is reasonable that these high-risk patients could be benefited from extended DAPT by preventing thrombotic complications in long-term prognosis (30). Therefore, we compared the clinical outcomes of prolonged DAPT (>1 year) vs. shortened DAPT (≤ 1 year) in patients with elevated Lp(a) levels who underwent PCI with DES.

Potentially the important finding of our study was that prolonged DAPT reduced the risks of MACCE, all-cause mortality, cardiac mortality, and definite or probable ST at 2.4 years in patients with elevated Lp(a) levels after PCI with DES. Additionally, the incidence of clinically relevant bleeding was similar between DAPT> 1-year and DAPT ≤ 1-year groups. Our previous study reported that DAPT continuation beyond 1 year offered a substantial reduction in ischemic cardiovascular events without apparent increase in clinically relevant bleeding risk compared with ≤ 1-year DAPT in patients with ESC/EACTS guideline-endorsed HTR features (19). Of note, patients with CAD and elevated Lp(a) levels had been demonstrated to be at heightened risk for ischemic events, and considered as an important group with high ischemic risk. The current study found that these patients can benefit from prolonging DAPT duration after PCI with DES at a median follow-up of 2.4 years. In this setting, Lp(a) levels might be a new important consideration when deciding the duration of DAPT after PCI in the future.

However, our study presented several limitations. First, this is a single-center, non-randomized study, and it is limited by unbalanced baseline characteristics and selection bias. Actually, the duration of DAPT was not predefined but was individualized by physician discretion. Although rigorous multivariable-adjusted analysis and IPTW analysis were performed, it was hard to control all the confounding factors and eliminate the selection bias. Nonetheless, our findings reflect the real-world practice in that treatment was tailored to individual patient risk, as is recommended in current guidelines. Second, DAPT regimen in our study was based on the use of clopidogrel and aspirin; therefore, the clinical impact of DAPT > 1 year with more potent P2Y_12_ inhibitor plus aspirin in CAD patients with high Lp(a) concentrations after PCI remains unclear. Third, Lp(a) was determined as mass concentration other than particle concentration, thus variations of apo(a) size between assay calibrators and patients' samples might overestimate or underestimate the real level of Lp(a). Luckily, a Lp(a) protein validated standard was used to calibrate the examination, along with linking the results to the World Health Organization/International Federation of Clinical Chemistry and Laboratory Medicine International Reference Reagent, making the assay relatively isoform independent. Fourth, the follow-up time should be extended to better specify the effect of prolonged DAPT on long-term outcomes.

## Conclusions

In patients with elevated Lp(a) concentrations who were event-free at 1 year after PCI with DES, prolonged DAPT (>1 year) reduced ischemic cardiovascular events, including MACCE, all-cause mortality, cardiac mortality, and definite/probable ST, without increase in clinically relevant bleeding risk compared with ≤ 1-year DAPT. Further well-designed, randomized trials are needed to confirm these findings.

## Data Availability Statement

The raw data supporting the conclusions of this article will be made available by the authors, without undue reservation.

## Ethics Statement

The studies involving human participants were reviewed and approved by the Ethical Committee of Fuwai Hospital, National Center for Cardiovascular Diseases. The patients/participants provided their written informed consent to participate in this study.

## Author Contributions

KC, LF, and KD: study design and interpretation of results. DY, CZ, WS, HW, LJ, LF, and KD: angiography review and patient enrollment. DZ, CS, KC, and H-YW: data collection. KC, WS, HW, and LJ: data analysis. KC and H-YW: preparation of manuscript. KC, H-YW, LF, and KD: revision of manuscript. All authors contributed to the article and approved the submitted version.

## Funding

This study was funded by CAMS Innovation Fund for Medical Sciences (CIFMS) (2021-I2M-1-008) and Beijing Municipal Health Commission-Capital Health Development Research Project (2020-1-4032). Sponsors were not involved in study design; in the collection, analysis, and interpretation of data; in the writing of the report; and in the decision to submit the article for publication.

## Conflict of Interest

The authors declare that the research was conducted in the absence of any commercial or financial relationships that could be construed as a potential conflict of interest.

## Publisher's Note

All claims expressed in this article are solely those of the authors and do not necessarily represent those of their affiliated organizations, or those of the publisher, the editors and the reviewers. Any product that may be evaluated in this article, or claim that may be made by its manufacturer, is not guaranteed or endorsed by the publisher.
